# Commentaries on *the Organon*

**Published:** 1892-09

**Authors:** B. Fincke

**Affiliations:** Brooklyn, N. Y.; Bureau of Homœopathic Philosophy, I. H. A.


### COMMENTARIES UPON *THE ORGANON*, §§ 7–17, 29, 64, 288. THE DYNAMICAL, SPIRIT-LIKE LIFE-FORCE.

B. FINCKE, M. D., BROOKLYN, N. Y.

(Bureau of Homœopathic Philosophy, I. H. A.)

The first sections of *The Organon* form the inductive part of the essential doctrine of homœopathics. Hahnemann starts from the facts which he has observed, and rises to the conception of disease being the totality of the symptoms as the outwardly reflected picture of the internal essence (*Wesen*) of disease; *i. e.*, the suffering of the life-force (§7). Then he proceeds to the pivot of the whole science of Homœopathics, the statement of the existence of a life-force which rules and controls the material sound organism in all its parts (§ 9). What he says about it in the few sections following is so clear that only a mere opposition can find in it faults which do not exist. There is a natural aversion of the practical physician to enter upon explanations of facts which he calls a theory, and to which he objects on that ground, as impractical for healing. This feeling was expressed in the preface to the first American edition of *The Organon*, which you all know: "It is the genuine Hahnemannian spirit to totally disregard all theories, even those of one's own fabrication, when they are in opposition to the results of pure experience." It is the ever-ready argument brought against Hahnemann himself or anybody, when he attempts to explain facts which cannot be controverted, to the disadvantage of understanding what these facts mean. If facts are stubborn things, they certainly need the more examining and explaining, and, if this is not possible, pointing out where they seem to clash with accepted ideas. To this famous quotation, which can be turned to account in both ways, for and against, is added the modest utterance of Hahnemann himself in § 28, where in regard to the natural law mentioned in § 26 he confirms its universality as a fact, but at the same time says that it is of little consequence to explain how this may be, and he attached little value to an effort to attempt it. But in spite of this, in the next section he goes bravely on to give the best explanation which can be given, and which indeed, as he says, is the most probable, since it is founded upon nothing but premises of experience. Nay, he continues in the following sections, and especially §§ 63 *seq.* to lay down a supposed doctrine of first and after-action which is a deduction not justified by the facts, and by the induction from facts, and by no means clear, as has been shown in the Commentaries which I had the honor to present to the International Hahnemannian Association at the last two meetings. It is the Hahnemannian spirit to disregard theories when in opposition to facts. This may excuse the attempt to show the incorrectness of § 64, though it seems to be almost a sacrilege to the mind which looks up to Hahnemann as inspired in everything that he put forth. As honorable as this sentiment is, it must be guarded against, for with riper judgment and new experience some of the ideas must be refuted which were held when beginning to ascend the ladder of wisdom. Piety to a beloved teacher must not upset truth when it goes against his teaching.

There is another discrepancy which must be pointed out because it has been used by some antagonist to, as it were, commit the Hahnemannians to a felony as if they did not cure as Hahnemann did. In §§ 284 and 285 and notes, Hahnemann gives a computation of the amount of medicine-matter contained in his potencies. Whilst in § 16 he claims that only spirit-like (dynamical, virtual) disturbing forces of the serviceable medicines can affect the likewise spirit-like (dynamical) life-force in a spirit-like (dynamical) manner, afterward in all his dealings with the healing potencies he disabuses himself of this view, for he falls back upon the materiality of his potencies. In § 155 he speaks of comminution of the dose, of the small, the least possible dose, which implies that the largest dose is the dose of the crude substance which is diminished in the amount of its constituent parts. In the *Chronic Diseases* (vol. I), he asserts that insoluble substances like metals by triturating them centesimally three times, become soluble in a mixture of alcohol and water, and in the note to § 280 (*Organon*) he thinks that there must be something of the original substance in the thirtieth centesimal potency left. This plainly contradicts the postulatum in § 16. However, we need not dwell too much upon this discrepancy, because after all Hahnemann placed not a great value upon it as being only an explanation, but insisted upon it that by the manipulatiou which he recommended the medicinal power would be developed (potency, force-development) so as to be perfectly in keeping with what he had said before in the mentioned initial sections of *The Organon.* If, therefore, the objectors spoken of above will overthrow the Hahnemannian theory and explanation about the first and after-action, as in § 64, and of the materiality of potencies higher than those in which any matter can be detected, they are welcome to the Hahnemannian spirit to demolish the theories not consistent with facts even of one's own making. But at the same time they must be reminded that where the explanations coincide with the facts they must abide by the theory explained until something better is offered. For in apposition to the above-quoted celebrated negative sentence a positive one is to be added, as the reverse of it which likewise is true. It is the genuine Hahnemannian spirit to accept a theory which is found to be true in its premises and logical in its conclusions, and therefore is in unison with pure experience and incontrovertible facts.

Such a theory we find in the sections dealing of the life-force which Hahnemann calls spirit-like, dynamical. But as soon as the adjective "spirit-like" is uttered, the empiric gets on his hind legs and asks, "What is spirit? To know what is spirit-like I must know first what is spirit, for if that is sufficiently explained the application to the life-force follows of itself." Now this, on the face of it, appears to be a very justifiable objection to the term which Hahnemann used, and it looks quite philosophical, because it seems to demand the premise to be placed upon a firm basis. But at the same time it looks very much like a child reaching with his little hands toward the moon which dazzles his eyes. Does the moon cease to exist because the baby cannot catch it? Thus it is with a conception like that of spirit, which, if a comparison is allowed, has a much greater range than the moon itself. For a definition of what is spirit is much easier asked than given. Webster has fifteen definitions. The objector can at least select from them what in the Hahnemannian life-force may be like it. Most of the definitions at any rate have the characteristic of refinement in contradistinction to crudity, of immateriality to materiality, of expansion to solidification, of activity to sluggishness, of motion to rest, of development to stagnation, of intellect to imbecility, of the psychical to the corporeal, of life to death, of immortality to mortality, etc.

To be honest, we all know without going into metaphysics what Hahnemann understood by spirit-like, and why should we by supercilious or skeptical objectors be driven into attempting the explanation and definition of spirit which no philosophic system so far has been able to give? Are the homœopathicians bound to supply the shortcoming of other sciences, as our friend on the other side has expressed it with regard to our high potencies that we have to overthrow the theory of physical science and put something better in its place? It cannot be denied that homœopathics has, indeed, made a powerful inroad into the accepted theories of not only the old school of medicine but also into those of the schools of exact sciences, and the time which Joslin predicted in 1850 may not be very far off, when he said, " It is the destiny of Homœopathy not only to effect a glorious revolution in the art of healing, but to lead to new views of the constitution of matter." Only the *short*-sighted homœopathician can try to save a remainder of materialism for our high potencies when he imagines there must be something of the original substance in the, say, one-hundred-thousandth centesimal potency, the efficaciousness of which is now sufficiently verified as to admit it as an incontrovertible fact. A sentence which I found in an old journal is worth quoting, because it clearly expresses the fact of the medicinal force residing in the crude substance as its vehicle, and being transferred by potentiation upon large quantities of inert vehicle. Dr. T. F. Allen wrote in the *New England Medical Gazette,* in July, 1870

"The efficacy of this (twentieth centes.) and higher potencies being, to my present mind, fully established, it follows that medicinal property is a force which can be isolated from material substance and be transmitted independent of it. What that force is and what are the laws which govern it are questions for extended scientific research." What here is predicated of the twentieth centesimal and higher potencies applies with equal force to the highest, the twentieth being removed from the twelfth limit only by eight potencies.

The homœopathician must acknowledge the fact that the physicist and materialist can find nothing material beyond the conventional twelfth centesimal potency. In spite of that point accepted by the materialist, the materialistically inclined homœopathician claims the infinite divisibility of the substance because he has convinced himself by experiment and experience of the efficaciousness of such a high potency as mentioned; for how could it act—he thinks—if there were not something left of the original substance from which the potency was derived? Something is left indeed, but it is not a molecule, nor even an atom which already parts company in the said twelfth cent. potency, but something else; something of that spirit-like nature which was held by the original substance serving as a vehicle for it, something of that spirit-like nature which Hahnemann attributes equally to the life-force of man and to that of the potencies. The physicist and materialist proves by mathematical computation the size of material atoms beyond which there can be no further division, and the chemist joins him in resigning infinite divisibility of matter to the grave. Is not that coming nearer to the fulfillment of the prediction of Joslin? True, if we had not our own Hahnemann we would be utterly lost when contemplating the dilemma in which we are placed by the utter inability to prove also by a plausible explanation from physical reasons the efficaciousness of our high potencies which is proved experimentally by application upon the healthy and sick as well as by Neural Analysis. Some of us have thought to find a safety-anchor in the molecular theory of physics but in vain, for our high potencies exceed any conception of minuteness of substance even if we would be inclined to admit the mentioned computations of eminent mathematicians to be true, as they undoubtedly are. Our potencies outstrip the theoretical radiant energy of matter and molecular motion of matter because the dynamides themselves, sunlight, magnetism, and electricity have been made into homœopathic high potencies and bottled up in our homœopathic vials. Besides, there is a general philosophical objection to the molecular theory which makes the molecules act of their own accord. It is said in the text-book that molecules are powerless to change their own condition, which is correct according to the first law of motion concerning the inertia of matter. At the same time it is believed that the molecules of every body are in motion always though no molecule can in any way change its own velocity or direction. But it leaves us in the dark where that molecule acquires the motion in which it *always is.* If the molecule, as the terms implies, is a synonym for an exceedingly small mass of matter, it is still a mass subject to the laws of motion, and cannot move except it be acted upon by a force. This is the fatal begging the question in this theory, that a force acting in the molecule is assumed, which is not proved and must first be proved to be acceptable. Another question arises in regard to sunlight which is supposed to be the undulation of an ethereal medium whose energy produces vision. Here again the premise that light passes through a vacuum is contradicted by the unproved assumption that a thin elastic substance called ether fills that space or the supposed vacuum, and therefore the premise cannot be accepted. On the contrary, the old Newtonian idea of light being an emanation of the sun gains new ground. For by Spectral Analysis we know that the Frauenhofer lines are caused by the incandescence of various substances as found on the earth when we burn them in a hydrogen flame in a dark room and throw the spectrum on a screen. We then observe the bright lines of the substances corresponding to the lines in the sun-spectrum and see them converted into dark Frauenhofer lines when we turn the sun-spectrum upon it. These substances then can come from no other source than the sun, because neither the intervening space nor the atmosphere is made accountable for them, but only the vibration of the ether imparted by the energy of the sun. It is not our object extensively to criticize the molecular theory, but only to show some inconsistencies in it for the purpose of protecting ourselves from the pretensions of materialistic philosophers who try to foist the molecular theory upon our potentiation, because we occupy an entirely different standpoint, which is the Hahnemannian, and has not yet found attention in the scientific world. Only this much may be added, that the philosophy of physics and chemics is by no means fundamentally antagonistic to it, for the underlying principles of these sciences are essentially founded upon the same general dynamic principle which is distinctly expressed in the definition of physics in the text-book, that it is the science of matter and force. Nay, these great scientific departments might without inconsistency adopt all the primary teaching of homœopathics as laid down in the first thirty paragraphs of *The Organon,* without in the least imperilling the truth and wealth of these sciences, but rather enriching them and giving them a sounder foundation.

Their own facts, which they have ascertained and which have brought our age to the highest point of technical perfection ever since sciences have been cultivated, stand all the same unshaken and corroborate the three grand laws of motion which govern them all as they do everything in existence, and which also our homœopathic science acknowledges as fundamental. To assign to matter all phenomena to the exclusion of that which enables it to appear, and blinding the eye to its inertia, assuming it to be the omnipotence governing the world would be as unphilosophical as making abstract force the almighty agent for all the varying occurrences in the universe. Our experience cannot disengage our mind from the omnipresence of matter in the most varying quantities, as long as we ourselves are incased in a material body. Just as we, in our high potencies, which are incased in the material body of Sugar of Milk, Alcohol, or Sugar Globules, recognize healing forces which, when properly applied, will change disease into health, so we cannot fail to recognize in our own material body a force which stands as the resultant of all the forces of the materials of which the organism is composed. This is a matter of experience and observation, and cannot be gainsaid, and aside of all metaphysical speculations it is as tangible as a pound of iron in your hand. It remained for such a perverted intellect as the physico-chemical school revealed when it threw out of physiology the fact of a life-force governing, regulating, and controlling the organism, and putting in its place the imagined properties of matter on the strength of an uncertain molecular theory. It can only be excused by the observation that "*non omnia possumus omnes,*" that one may be proficient in one department, and reach as high as heaven, and in another cling to the ground, unable to lift his wings above the requirements of logic.

When Hahnemann speaks of a life-force, he does not mean to go beyond the simple fact that such a force exists. He does not say where it comes from nor where it goes to. True to his practical nature and fortunate for his doctrine, he abides by his inductive philosophy, which teaches him that such a life-force is immanent in every even most insignificant part of the body as well as in the whole, and proving itself as such at any time by sensation. The least touch in any part of the body proves that it is there, and every departure from health is indicated by symptoms which for their apparent insignificance may often be overlooked, but severe or slight, they all verify the ever-present activity of the life-force. Some years ago I went to see a young hippopotamus, which was already as large as a cow. Its skin was of a reddish cast, as that of an infant, but it was thick and hard, looking very much like the surface of a pine-apple. It was lying quietly looking before itself. I was curious to know whether it would notice a slight touch at the hind part of its body, and just touched the skin very slightly with a little stick. It immediately turned its head around, and showed that its life-force was keen enough to notice that insignificant contact. You say it was its sensation. Yes, that is the abstract of the fact. But there was that in its huge body which communicated the sensation to the consciousness of the animal, and made it turn its head around, and that was the automatic, instructive life-force of the creature. Hahnemann does not call this force the soul or spirit. This would have led him on debatable ground, for here religion would have come in with its necessary creeds, and set the much varying opinions by their ears, which would have marred the practical teaching of his homœopathics. There is nobody in the world claiming any thinking faculty but must perceive that there is that in man which makes him think about his impressions, makes him feel and will and act. Some call it soul and some spirit, but whatever it may be called, it is certainly something different from matter, and under conditions and laws which have nothing more to do with it than that it serves the high purpose for which it is designed (§ 9). If this is admitted, the question naturally arises, how this spiritual organism—as it might be called in contradistinction to the material organism—in the body is connected with the matter used in building up and preserving it during its lifetime. This is a perfectly legitimate question, and its solution belongs to the science of medicine which made an enormous progress when Hahnemann proclaimed the dynamicity of diseases, and claiming for their healing medicines prepared in such a manner as to meet them on their own plane of dynamicity.

This connecting link between the body and the spirit, between the nether and the upper world consists in that dynamic organ which he called the spirit-like life-force. The means by which this authority performs its functions are the various organic systems, the working of which is the proper subject of investigation by that particular important branch of medicine, physiology, and its special department, biology. Hahnemann, by his invention of potentiation, has given us a new view of the world, because he infused life into everything, and thus animated matter so that it can serve as the vehicle for the forces with which it has been endowed by the Creator, and can enter into the various inorganic and organic forms which are around us. To be sure there is no spirit nor soul in the grain of sand which our foot treads upon, but there is that in it which constitutes it a grain of sand. And to this belongs the medicinal force which it carries and yields upon potentiation as part of its own life-force and individuality so that it can be useful in healing diseases to which in its individuality it corresponds. The higher life-force of man is acted on by this lower life-force of a grain of sand, and though not themselves spiritual—*i. e.*, endowed with reason—Hahnemann calls them, on account of their dynamicity spirit-like with perfect justice, and no man or woman need stumble over this expression when reading *The Organon*, because it says just what it means and no more, and that is sufficient.

