# Correction

**Published:** 1893-01

**Authors:** 


Correction.—In the September number, page 382, first line, for "instructive" read INSTINCTIVE. A fatal error.

